# Subcutaneous injection-induced cellulites

**DOI:** 10.1051/bmdcn/2017070206

**Published:** 2017-06-14

**Authors:** Kao-Chi Cheng, Po-Tsung Huang, Chiu-Shong Liu, Wen-Yuan Lin

**Affiliations:** 1 Department of Family Medicine, China Medical University Hospital Taichung 404 Taiwan; 2 Department of Family Medicine, College of Medicine, China Medical University Taichung 404 Taiwan

**Keywords:** Subcutaneous, Cellulitis

## Abstract

In the hospice ward where patients are in the terminal stages of cancer, it is common practice to give them a subcutaneous injection of pain relievers to reduce their pain and make them more comfortable. Most of these patients are elderly and have low blood pressure or poor veins, which often makes it difficult to inject them because of the calcification at previous injection sites. Thus, subcutaneous injections are a convenient way to maintain analgesia and patient comfort.

Our patient, a 73-year-old aboriginal woman, was diagnosed with gastric adenocarcinoma and peritoneal carcinomatosis in March, 2004. While she was in the inpatient hospice ward, a subcutaneous injection site became infected and localized cellulitis developed. The patient’s quality of life began to decline and her hospice stay was lengthened due to these complications. This case is offered as a reference case of subcutaneous injection complications encountered by elderly patients in hospice care.

## 1. Introduction

Among hospitalized patients, intravenous injections are very common. Because many patients with terminal-stage cancer have unstable and confused states of consciousness, local cellulitis or phlebitis can result at intravenous injection sites.[1, 2] However, due to the diligence of medical professionals and lay patient caregivers, the rate of these well-documented complications has dropped to its present level.[3] Nonetheless, among inpatients in the hospice ward, subcutaneous injections are common procedures, but there are few data documenting complications from them. This case is offered as a reference case of subcutaneous injection complications encountered by elderly patients in inpatient hospice care.[3, 4] One study showed that subcutaneous fluid delivery is an effective method of rehydration and of opioid administration, and can prevent the need for intravenous catheterization and consequently hospitalization. It is a simple procedure to initiate, safe, less distressing to the patient, and does not predispose the patient to intravenous related infections.[5]

## 2. Case report

The patient was a 73-year-old aboriginal woman with a medical history of cardiovascular disease and bilateral knee degenerative arthritis treated with bilateral total knee replacement. She was diagnosed with gastric adenocarcinoma and peritoneal carcinomatosis in March, 2004. She underwent palliative subtotal gastrectomy with Bn anastomosis and feeding jejunostomy in April of the same year. During rehabilitation, her doctor informed her about the limitations of chemotherapy and radiotherapy, and suggested that she consider accepting hospice care. She and her relatives decided to accept hospice care in order to promote the quality of her remaining life.

The patient was admitted to the hospice ward due to abdominal pain and nausea with vomiting in May, 2004. During hospice admission, she experienced 1) post jejunostomy wound care issues, 2) cancer related pain, 3) cachexia, and 4) intestinal obstruction. All of these complications were promptly treated and resolved.

**Fig. 1:**
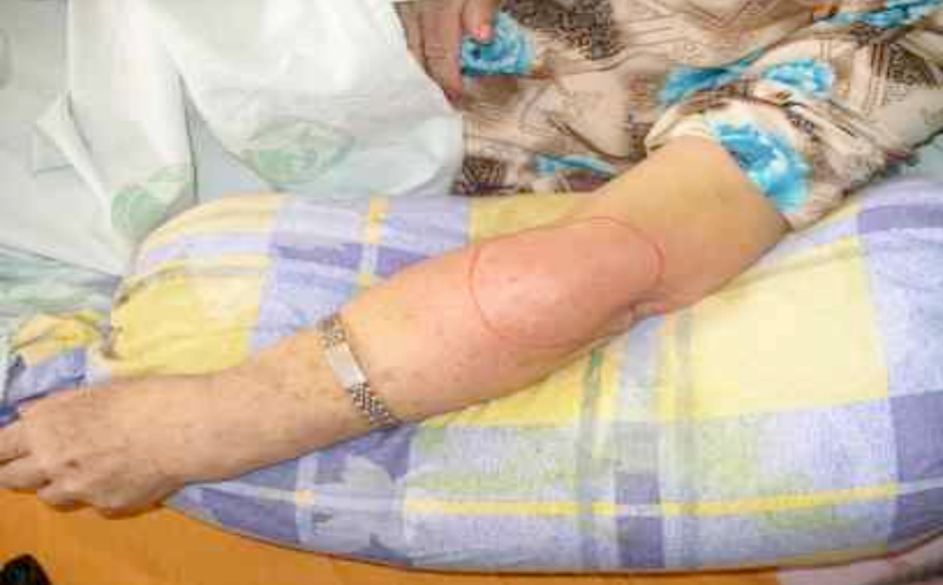
Before management:highly suspect subcutaneous local cellulitis.

The nursing staff noticed that the patient often scratched her subcutaneous injection sites with her hands due to localized itching. Even after instruction by the nursing staff not to scratch the injection sites, the patient scratched them unconsciously. When she developed tenderness of the subcutaneous injection site on her left forearm in June, 2004, oral cephalexin (250 mg) was administered four times daily. Two days later, local redness with tenderness intensified and she developed a fever of 38°C with chills (Fig. 1). A complete blood count showed an elevated white cell count and the erythrocyte sedimentation rate was also elevated. Blood cultures and Gram stains revealed negative findings. Her antibiotic was switched to intravenous prostaphylline 1 g every 6 hours due to a high suspicion of local cellulitis.

**Fig. 2:**
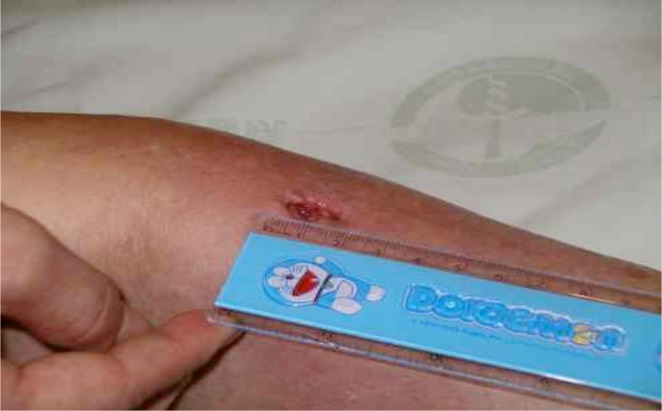
After management: pus formation with dischagre.

Two days later, the patient’s fever subsided, and the infected site had local pus formation and discharge. While antibiotic therapy was not changed, incisional drainage was performed (Fig. 2). The discharge was cultured and grew Klebsiella pneumoniae.[1, 2, 6, 7] Two stitches were used to close the wound and the dressings were changed daily. The wound drainage became clear and healed well. The patient was discharged on June 28 to hospice home care.

## 3. Discussion

In the hospice ward, it is very common to administer many different types of drugs *via* a subcutaneous injection instead of intravenously[3]. Intravenous injections are often complicated by local infiltration, infection, and phlebitis. After a patient’s condition has stabilized and intravenous medications are no longer necessary, we prefer to give medications by the subcutaneous route for convenience and for home care. Among patients in the terminal stage of cancer, most are elderly and have low blood pressure or veins that are often difficult to inject because of longterm treatment inducing calcification, or else the veins are hard to find. For these reasons, it is common and convenient to administer medications *via* a subcutaneous route.

In the last decade, hypodermoclysis has been claimed to be an easy and safe method of fluid replacement in non-emergency situations, especially in elderly and terminally ill patients where intravenous access may be difficult and the procedure may need to be maintained for weeks.[8–13] For example, subcutaneous and intravenous fluids have been shown to achieve equivalent biochemical rehydration of stroke patients.[14] Hypodermoclysis is also suitable for the delivery of opioid infusions for relief of pain in cancer patients.[15]

There is little data on the complications of subcutaneous injection in the medical literature, perhaps because medical personnel believe that intravenous injections are more often complicated by cellulitis than subcutaneous injections are.[7, 16] Accordingly, some medical personnel may not look for complications such as cellulitis associated with subcutaneous injections. Intravenous injection sites are changed once every 3 days, while subcutaneous injection sites are changed once per week. It is our practice to inspect the injection site for redness and promptly change sites if it is deemed unsuitable for injection.

Sometimes patients in the hospice ward for terminal cancer will scratch the injection site due to localized itching.[7, 16] Many times, this occurs during periods of unstable or drowsy consciousness. Then, the small epidermal wound becomes infected, causing local cellulitis. Most hospice patients are elderly or immunocompromised, which causes the condition to worsen, influencing their treatment and lengthening their hospital stay. We trust that this case report will heighten awareness of the seldom-mentioned complications of subcutaneous injection sites among elderly hospice patients.
